# Illness perceptions of obstructive sleep apnea (OSA) and intention of performing clinical diagnostic examination for OSA in Chinese older adults

**DOI:** 10.1186/s12889-025-23278-y

**Published:** 2025-07-02

**Authors:** Yanqiu Yu, Joyce Hoi-Yuk Ng, Mason M. C. Lau, Joseph T. F. Lau

**Affiliations:** 1 Department of Preventive Medicine and Health Education, School of Public Health, Fudan University, Shanghai, China; 2 Jockey Club School of Public Health and Primary Care, The Chinese University of Hong Kong, Hong Kong, China; 3 Public Mental Health Center, School of Mental Health, Wenzhou Medical University, Wenzhou, China; 4 Zhejiang Provincial Clinical Research Center for Mental Disorders, The Affiliated Wenzhou Kangning Hospital, Wenzhou Medical University, Wenzhou, China; 5 Center for Health Behaviours Research, The Chinese University of Hong Kong, Hong Kong, China

**Keywords:** Obstructive sleep apnea, Illness perception, Illness representation, Older people, Common sense model, Chinese

## Abstract

**Background:**

Obstructive sleep apnea (OSA) is highly prevalent, under-detected, but preventable among older adults. The Common Sense Model of Illness postulates that illness representations are associated with coping and behavioural outcomes. This study investigated the associations between illness representations of OSA and behavioral intention of clinical diagnostic examination for OSA (BICDE-OSA) under two conditional/unconditional situations, stratified by high-risk versus low-risk OSA.

**Methods:**

A random population-based telephone survey interviewed 945 Chinese people aged ≥ 50 years without OSA diagnosis in the Hong Kong general population from February to November 2021.

**Results:**

The prevalence of unconditional BICDE-OSA based on the participants’ current sleep situation was only 5.3%; that of conditional BICDE-OSA based on the hypothetical situation of having OSA was 54.3%. Significantly higher prevalence of conditional BICDE-OSA was found in the high-risk than the low-risk of OSA groups (64.4% versus 52.9%; *p* < 0.05); no significant between-group difference in unconditional BICDE-OSA was found. Illness representations of timeline chronic, timeline cyclical, consequences, and emotional representation were significantly associated with both conditional/unconditional BICDE-OSA (adjusted for background factors); illness coherence was significantly associated with conditional BICDE-OSA but not with unconditional BICDE-OSA.

**Conclusions:**

High proportions of older people did not indicate conditional/unconditional BICDE-OSA, possibly resulting in low detection and treatment rates. As OSA was prevalent in the study population and the unconditional group showed a much higher prevalence of BICDE-OSA than the conditional group, the prevalence of BICID-OSA might increase if the high-risk people are made aware of their risk. Interventions modifying illness representations may be considered.

## Introduction

Obstructive sleep apnea (OSA) is a severe health threat among older people. A systematic review reported a high prevalence of moderate-to-severe OSA among males and females aged ≥ 50 [1]. The prevalence of mild-to-severe OSA increased with age [2]. As a high proportion of those aged ≥ 50 years are suffering from mild-to-severe OSA [2], the present study targets this age group. Meta-analyses and reviews have shown that OSA is highly detrimental to physical health [3], mental health [4], problems in daily life, and inter-personal relationships [4, 5].

OSA is, however, highly treatable. A meta-analysis reported that treatments such as non-invasive continuous positive airway pressure (CPAP) therapy [6, 7] effectively reduce symptoms and harms of OSA. Yet, a majority of the OSA patients remain undiagnosed and untreated. For instance, the OSA detection rate was only 15% according to a U.K. national report [8]. Several cross-sectional studies reported that the determinants of taking up clinical diagnostic examination for OSA, in general, included obesity [9], sleep conditions (e.g., daytime sleepiness and sleep efficiency) [9–11], and awareness of high-risk OSA status due to screening tests [10–13]. In addition, several qualitative studies have reported that facilitators of performing clinical diagnostic examinations for OSA included adequate knowledge, tangible experiences of consequences of OSA, and support/suggestions from important others (e.g., spouses and healthcare workers) [14–19], while barriers included a lack of awareness, inadequate knowledge, psychological factors (e.g., personality traits and fears of the test/device), cost, and service system (e.g., healthcare providers’ misdiagnosis and logistics) [14, 16–23]. Perceptions of OSA seem to be an under-researched topic. Notably, perceptions of illnesses are known predictors of health-related behaviors, including health-seeking behaviors [24, 25]; they have also been used as key components of health education and promotion programs [26, 27]. More research in this regard is hence needed.

This study is grounded on Leventhal’s Common Sense Model of Illness (CSM) [28], which has widely been applied to understand determinants of health-seeking behaviors related to physical diseases (e.g., heart failure) [24] and mental problems (e.g., depression) [25]. It postulates that behavioral coping responses and outcomes regarding an illness are determined by related illness perceptions. Illness perceptions, namely illness representations, refer to how diseased/non-diseased people think and feel about an illness [28]. In this study, the illness is OSA; the behavioral response is the behaviour intention of performing a clinical diagnostic examination for OSA (BICDE-OSA). Illness representations consist of two parallel yet interrelated sets of cognitive and emotional responses (i.e., cognitive representations and emotional representations) to the stimulus of a disease condition [28]. Cognitive illness representations comprise eight constructs: [1] Identity (symptoms) [2], Timeline chronic (perceived duration of an illness) [3], Timeline cyclical (perceived recurrence of an illness) [4], Consequences (perceived negative impacts of an illness) [5], Personal control (how much an illness can be controlled by personal behaviors) [6], Treatment control (perceived treatability of an illness) [7], Illness coherence (knowledge or understanding regarding an illness), and [8] Cause of the illness. The emotional representation construct refers to negative emotional responses to an illness (e.g., anxiety and anger). Levels of illness representations are commonly measured by the 38-item Revised Illness Perception Questionnaire (IPQ-R) [29]. A Chinese version of the IPQ-R for OSA (29-item) has been validated [30] and was used in this study. Only two previous studies have examined the associations between illness representations of OSA and the use of CPAP [31] and treatment adherence [32] among diagnosed OSA patients. Both studies, however, used the 8-item Brief Illness Perception Questionnaire of OSA, of which the reliability and validity had not been examined, but not the full version. To our knowledge, no study has investigated the associations between illness perceptions of OSA and performing a clinical diagnostic examination for OSA among undiagnosed high-risk individuals.

Given the background, this study investigates the prevalence of BICDE-OSA and its associations with illness representations of OSA among Chinese adults aged ≥ 50 years who have not been diagnosed with OSA in the Hong Kong general population. Behavioral intention is a known and strong predictor of actual behavior and part of the commonly used Theory of Planned Behavior (TPB) [33], which postulates that attitude, subjective norm, and behavioral control determine intention, which in turn determines the behavior. It is hypothesized that the seven domains of illness representations of OSA (i.e., Timeline chronic, Consequence, Personal control, Treatment control, Illness coherence, Timeline cyclical, and Emotional representation) would be positively associated with BICDE-OSA. To better inform tailor-made health promotion, the analyses are stratified by the participants’ risk status related to OSA (high risk versus low risk).

## Methods

### Participants and data collection

A population-based random telephone survey was conducted in the Chinese Hong Kong general population aged ≥ 50 years from February 5th to November 10th, 2021. Inclusion criteria were [1] ≥ 50 years old [2], Hong Kong residents, and [3] Chinese-speaking. Exclusion criteria included those who [1] had never heard of OSA and [2] had an OSA diagnosis by a doctor or self-reported having performed an OSA clinical examination without an OSA diagnosis. Telephone numbers were randomly drawn from the most updated residential fixed-line telephone directory. The interviews were conducted between 5 and 10 pm to avoid over-sampling non-working individuals. Unanswered telephone calls were given at least three attempts. The eligible household member whose birthday was closest to the survey date was interviewed. Appointments were made if necessary. Trained interviewers briefed the participants about the study, sought their verbal informed consent, and signed a form pledging the completion of the required procedures. No incentives were given to the participants. Participants could quit at any time without being questioned.

Out of the 1,720 eligible contacts made, 955 participants completed the interview (response rate: 955/1,720 = 55.5%); 10 participants were further removed due to missing responses in variables related to the risk levels of OSA. The effective sample size was 945 in this study.

### Patient and public involvement

No patients were involved in this study.

### Ethical statement

This study was approved by the Survey and Behavioral Research Ethics Committee of the Chinese University of Hong Kong (No. SBRE-20-498).

### Measures

#### Background information

Socio-demographic information was collected, including sex, age, educational level, and marital status.

#### Unconditional and conditional BICDE-OSA

The participants’ BICDE-OSA (next 12 months) was assessed by two items covering two scenarios: (a) the behavioral intention based on the participant’s current sleep situation (i.e., unconditional BICDE-OSA), and (b) the behavioral intention under a hypothetical situation that the participants perceived currently having OSA (i.e., conditional BICDE-OSA). The 5-point Likert scales (1 = definitely no to 5 = definitely yes) were recoded into two binary variables (Yes: definitely yes/likely yes versus No: half-half/likely no/definitely no) to indicate the presence of unconditional and conditional BICDE-OSA.

#### Risk levels of OSA

The 11-item Berlin Questionnaire [34] was used to assess participants’ risk level of OSA, which comprises three risk categories: (a) snoring severity, (b) excessive daytime sleepiness, and (c) history of high blood pressure or obesity (defined as BMI ≥ 30 kg/m^2^). Participants were classified as having a high risk of OSA if two or three categories indicated some symptoms/risk factors; the others were classified as having a low risk of OSA [34]. Following some previous telephone surveys, weight and height were self-reported in this study [35–37]. The questionnaire has been validated in Chinese [34, 35, 37] and has been used in many regions, including Hong Kong [38].

#### Illness representations of OSA

The validated 29-item Chinese version of the Revised Illness Perception Questionnaire of OSA (IPQ-R-OSA) was used to assess the illness representations of OSA [30]. It included seven domains of illness representation, i.e., Consequences, Timeline chronic, Timeline cyclical, Illness coherence, Emotional representations, Personal control, and Treatment control. Like some previous studies [30, 39, 40], the two domains of Identity and Cause were not included in the IPQ-R-OSA. The seven subscales were measured using 5-point Likert scales (1 = strongly disagree to 5 = strongly agree). The subscale scores were divided by the number of items to keep the comparable range within 1 to 5 (midpoint = 3.0). Higher scores indicated higher levels of the corresponding constructs (e.g., more severe emotional representations). The Cronbach’s alpha of the seven subscales ranged from 0.6 to 0.8 in this study.

### Statistical analysis

The Chi-square test and t-test were conducted to test the between-group differences. Univariable logistic regression analysis was conducted to test the individual associations between the background factors (sex, age, educational level, and marital status) and conditional BICDE-OSA. Multivariable logistic regression analysis was conducted to test the individual associations between seven domains of illness representations of OSA and conditional BICDE-OSA, adjusted for potential confounders of the above four background factors. These background factors were treated as confounders based on their associations with both illness representations and health-seeking behaviors [41–43]. Collinearity diagnostics were also performed; two indicators and cut-off values of variance inflation factor (VIF) > 5 and tolerance < 0.1 were used to indicate the presence of collinearity among the background factors. In addition, the analyses were stratified by the high-risk and low-risk status. As only 3.8% (*n* = 5) and 5.5% (*n* = 45) of the participants at high-risk and low-risk of OSA reported a higher level of unconditional BICDE-OSA, respectively, the logistic regression analysis was conducted for only the outcome of conditional BICDE-OSA, but not unconditional BICDE-OSA due to the small number of cases with intention compared to the number of parameters. Statistical analysis was conducted by using SPSS 26.0. Statistical significance was denoted by two-tailed *p*-values < 0.05.

## Results

### Descriptive statistics

In Table 1, over half of the participants were female (66.9%). About one-third were aged > 70 years (35.3%); 15.3% had received tertiary education or above; 81% were currently married. According to the Berlin questionnaire, 14.0% and 86.0% of the participants were classified as having high risk and low risk of OSA, respectively. The prevalence of high-risk OSA was 18.5% for males and 11.7% for females (*p* = 0.004). Age, marital status, and education level were not statistically associated with the risk of OSA (Table 1).

**Table 1:** Descriptive statistics

	All (*n* = 945)			High risk of OSA (*n* = 132)			Low risk of OSA (*n* = 813)		*p* of χ^2^
	*n*		%	*n*	%		*n*	%	
**Background factors**									
Sex									
Male	313		33.1	58	43.9		255	31.4	0.004
Female	632		66.9	74	56.1		558	68.6	
Age group									
50–60	309		32.7	48	36.4		261	32.1	0.570
61–70	294		31.1	40	30.3		254	31.2	
Over 70	334		35.3	42	31.8		292	35.9	
Missing data	8		0.8	2	1.5		6	0.7	
Education level									
Secondary education or below	783		82.9	104	78.8		679	83.5	0.408
Tertiary education or above	145		15.3	25	18.9		120	14.8	
Missing data	17		1.8	3	2.3		14	1.7	
Marital status									
Others	170		18.0	19	14.4		151	18.6	0.451
Married	765		81.0	111	84.1		654	80.4	
Missing data	10		1.1	2	1.5		8	1.0	
**Behavioral intention**									
Unconditional BICDE-OSA									
No	826		87.4	111	84.1		715	87.9	0.486
Yes	50		5.3	5	3.8		45	5.5	
Missing	69		7.3	16	12.1		53	6.5	
Conditional BICDE-OSA									
No	430		45.5	47	35.6		383	47.1	0.014
Yes	515		54.5	85	64.4		430	52.9	

Note. OSA = Obstructive sleep apnea; BICDE-OSA = Behavioral intention of performing a clinical diagnostic examination for OSA. Unconditional BICDE-OSA refers to the BICDE-OSA in the future 12 months based on participants’ current sleep situation, while conditional BICDE-OSA refers to BICDE-OSA given a hypothesized situation that participant perceived currently having OSA

The prevalence of unconditional BICDE-OSA and conditional BICDE-OSA was 5.3% and 54.3%, respectively. The prevalence of unconditional BICDE-OSA did not significantly differ between the two groups having high and low risk of OSA (3.8% versus 5.5%; *p* = 0.486), while the prevalence of conditional BICDE-OSA of the high-risk group was statistically higher than that of the low-risk group (64.4% versus 52.9%; *p* = 0.014; Table 1).

The mean subscale scores of the illness representation constructs are shown in Table 2. The mean scores of Timeline chronic, Consequences, and Emotional representations were statistically higher in the high-risk group than in the low-risk group (Cohen’s *d* ranged from 0.18 to 0.35; all *p* < 0.05); the between-group differences in the other illness representations domains (Personal control, Treatment control, Illness coherence, and Timeline cyclical) were not statistically significant.

**Table 2:** Mean scores and between-group differences of illness representations of OSA

	All			High risk of OSA			Low risk of OSA			t	df	*p*	Cohen’s d
	Range	Mean	SD	Range	Mean	SD	Range	Mean	SD				
Timeline chronic	2.0-4.7	3.2	0.4	2.2–4.5	3.4	0.4	2.0-4.7	3.2	0.4	3.9	943.0	< 0.001	0.35
Consequence	1.3–4.8	2.8	0.5	2.0-4.2	2.9	0.5	1.3–4.8	2.8	0.5	2.6	191.0	0.010	0.23
Personal control	2.2–4.7	3.4	0.4	2.5–4.5	3.4	0.4	2.2–4.7	3.4	0.4	1.3	943.0	0.182	0.12
Treatment control	2.4–4.6	3.4	0.4	2.4–4.4	3.4	0.4	2.4–4.6	3.4	0.4	–0.2	943.0	0.816	0.02
Illness coherence	1.0-4.8	3.1	0.6	1.0-4.4	3.1	0.6	1.0-4.8	3.1	0.6	0.3	943.0	0.792	0.02
Timeline cyclical	1.0-4.3	3.0	0.3	2.0–4.0	3.0	0.3	1.0-4.3	3.0	0.3	1.8	943.0	0.072	0.17
Emotional representation	1.2-5.0	2.6	0.6	1.5-5.0	2.7	0.6	1.2–4.8	2.6	0.6	2.0	943.0	0.049	0.18

Note. OSA = Obstructive sleep apnea; SD = Standard deviation; *df* = degrees of freedom

### Factors of conditional BICDE-OSA

In the high-risk group, the associations between sex, age, education level and conditional BICDE-OSA were statistically non-significant (Table 3). Illness representations of Timeline chronic, Consequences, Emotional representation, and Timeline cyclical were significantly associated with conditional BICDE-OSA after adjusting for background factors (Table 4). Notably, collinearity diagnostics showed that VIF and tolerance of the four background variables ranged from 1.089 to 2.409 and from 0.415 to 0.966, respectively, indicating the absence of collinearity.

**Table 3:** Background factors of conditional BICDE-OSA (*n* = 945)

	Dependent variable = Conditional BICDE-OSA		
	All (*n* = 945)	High risk of OSA (*n* = 132)	Low risk of OSA (*n* = 813)
	ORc (95% CI)	ORc (95% CI)	ORc (95% CI)
Sex			
Male	Reference group	Reference group	Reference group
Female	1.07 (0.82, 1.41)	1.05 (0.51, 2.15)	1.12 (0.83, 1.50)
Age groups			
50–60	Reference group	Reference group	Reference group
61–70	1.16 (0.83, 1.62)	2.40 (0.91, 6.33)	1.06 (0.74, 1.51)
Over 70	0.38 (0.28, 0.53)***	0.66 (0.28, 1.53)	0.35 (0.25, 0.50)***
Missing data	0.62 (0.15, 2.52)	0.60 (0.04, 10.20)	0.62 (0.12, 3.14)
Education levels			
Secondary education or below	Reference group	Reference group	Reference group
Tertiary education or above	2.87 (1.92, 4.27)***	1.98 (0.73, 5.38)	3.02 (1.96, 4.67)***
Missing data	2.36 (0.82, 6.76)	1.25 (0.11, 14.24)	2.63 (0.82, 8.46)
Current marital status			
Others	Reference group	Reference group	Reference group
Married	1.64 (1.18, 2.30)**	1.08 (0.39. 2.96)	1.71 (1.20, 2.45)**
Missing data	5.07 (1.05, 24.57)*	0.58 (0.03, 10.86)	9.78 (1.17, 81.47)*

Note. OSA = Obstructive sleep apnea; BICDE-OSA = Behavioral intention of performing a clinical diagnostic examination for OSA; ORc = Crude odds ratio; CI = Confidence interval. ***, *p* < 0.001. **, *p* < 0.01. Conditional BICDE-OSA refers to BICDE-OSA given a hypothesized situation that participant perceived currently having OSA

**Table 4:** Associations between illness representations of OSA and conditional BICDE-OSA

	Dependent variable = Conditional BICDE-OSA					
	All (*n* = 945)		High risk of OSA (*n* = 132)		Low risk of OSA (*n* = 813)	
	ORc (95% CI)	ORa (95% CI)	ORc (95% CI)	ORa (95% CI)	ORc (95% CI)	ORa (95% CI)
Timeline chronic	1.31 (1.23, 1.39)***	1.26 (1.18, 1.34)***	1.22 (1.05, 1.42)**	1.23 (1.05, 1.43)*	1.32 (1.23, 1.41)***	1.26 (1.17, 1.34)***
Consequence	1.15 (1.10, 1.21)***	1.21 (1.15, 1.27)***	1.19 (1.03, 1.38)*	1.25 (1.07, 1.47)**	1.15 (1.09, 1.20)***	1.21 (1.15, 1.27)***
Personal control	1.17 (1.10, 1.24)***	1.09 (1.02, 1.16)*	1.03 (0.88, 1.20)	0.94 (0.79, 1.12)	1.19 (1.12, 1.27)***	1.11 (1.04, 1.19)**
Treatment control	1.06 (0.99, 1.13)	0.98 (0.91, 1.06)	1.19 (0.97, 1.45)	1.13 (0.92, 1.39)	1.04 (0.97, 1.12)	0.96 (0.89, 1.04)
Illness coherence	1.12 (1.07, 1.16)***	1.06 (1.01, 1.11)*	1.01 (0.90, 1.12)	0.96 (0.85, 1.09)	1.14 (1.09, 1.19)***	1.08 (1.02, 1.13)**
Timeline cyclical	1.04 (0.95, 1.14)	1.07 (0.98, 1.19)	1.38 (1.02, 1.87)*	1.68 (1.18, 2.38)**	0.99 (0.90, 1.10)	1.01 (0.91, 1.12)
Emotional representation	1.14 (1.10, 1.19)***	1.14 (1.10, 1.19)***	1.10 (0.99, 1.23)	1.13 (1.01, 1.26)*	1.15 (1.10, 1.20)***	1.15 (1.09, 1.20)***

Note. OSA = Obstructive sleep apnea; BICDE-OSA = Behavioral intention of performing a clinical diagnostic examination for OSA; ORc = Crude odds ratio; ORa = Adjusted odds ratio; CI = Confidence interval. ***, *p* < 0.001. **, *p* < 0.01. *, *p* < 0.05. Conditional BICDE-OSA refers to BICDE-OSA given a hypothesized situation that participant perceived currently having OSA. Adjusted models were adjusted for all background variables, including sex, age, education level and marital status

In the low-risk group (and also in the overall sample), tertiary education level and currently being married were positively associated with conditional BICDE-OSA while age > 70 years was negatively associated with conditional BICDE-OSA (see Table 3). The adjusted logistic regression analysis showed that illness representations of Timeline chronic, Consequences, Personal control, Illness coherence, and Emotional representation were significantly associated with conditional BICDE-OSA (Table 4).

## Discussion

About one-seventh of the sampled undiagnosed adults aged ≥ 50 in the Hong Kong general population were at high risk of OSA. Consistent with literature [1, 2], males were at higher risk than females. The non-significant age difference in OSA risk was unexpected. As diagnosed people were excluded from data analysis and the prevalence of OSA increased with age, it is plausible that more older people were excluded than younger people, which lowered the risk level of the sampled older people. It is, however, a limitation of this study that the age of the excluded cases was not recorded. The contention hence could not be tested in this study.

In this study, the prevalence of unconditional BICDE-OSA (given the participant’s current sleep situation) was very low. It was only 3.8% even in the high-risk group, implying that, given the present state, very few community-dwelling high-risk people had BICDE-OSA. The findings agree with the low OSA detection rate reported previously [8, 44], suggesting that the OSA detection rate might remain low in the absence of effective interventions. Notably, despite the low prevalence of unconditional BICDE-OSA, the prevalence of conditional BICDE-OSA was ten-fold higher than that of unconditional BICDE-OSA. This contrast suggests that many high-risk older people without BICDE-OSA might change their mind if they knew about their high-risk status. This contention of low-risk awareness was consistent with the relatively low subscale score of illness coherence, suggesting a relatively low level of understanding about OSA. Literature has also documented that lacking awareness and inadequate knowledge were barriers to performing clinical diagnostic examinations for OSA [14, 17, 18, 20].

Previous studies found a higher prevalence of behavior and intention regarding the uptake of health service utilization among female, younger, and better-educated participants [41, 42]. However, in the high-risk group, it was unexpected that conditional BICDE-OSA was not associated with these socio-demographics, implying that the low levels of conditional BICDE-OSA might prevail across socio-demographic groups. Interventions are hence warranted for all socio-demographic groups of those at high risk of OSA.

In the low-risk group, the prevalence of unconditional BICDE-OSA was only about 5%, while that of conditional BICDE-OSA increased sharply from 5 to 52.9%. The high prevalence of OSA among older people [1] implied that many low-risk individuals aged ≥ 50 might have a spouse or other family members with OSA. Family members (e.g., spouse) are in the best position to detect an individual’s OSA symptoms (e.g., snoring). Indeed, previous studies showed that family members are important sources of informational and emotional support to initiate performing clinical diagnostic examinations for OSA and subsequent adherence to CPAP [15, 16, 18–21, 45]. Thus, low-risk individuals (the general population) may play an important role in detecting OSA and increasing relevant help-seeking behaviors in the older population. Interventions promoting OSA examination may consider involving family members.

The findings suggest that modification of illness representations of OSA is potentially useful in promoting OSA examination, as such perceptions were associated with conditional BICDE-OSA. In general, the levels of the seven domains of illness representations of OSA were only at low to moderate levels; there is room for improvement. Although OSA evidently leads to severe physical and psychological harm [3, 4], the score of consequences in the high-/low-risk group was only 2.9/2.8 (lower than the midpoint of 3.0) and was significantly associated with conditional BICDE-OSA in both subsamples. Thus, even people at high risk of OSA were not aware of the potential severe harms of OSA, and they in general did not perceive OSA as threatening. In the literature, consequences have been associated with health-promoting behaviors (e.g., disease screening) [25]. As the severity of harm of OSA is well documented, interventions promoting OSA detection should incorporate such information.

OSA is a chronic disease condition [8]. In this study, timeline chronic was significantly associated with conditional BICDE-OSA in both high-risk and low-risk groups. Again, its mean values were not high in both groups. Furthermore, in the absence of treatment, OSA is unlikely to improve over time. Time cyclical was also a significant factor of conditional BICDE-OSA in the high-risk group. Thus, interventions should explain the chronic and possibly recurrent nature of OSA to the general population.

In the context of low perceived severity, it is not surprising that emotional representations showed a low level of 2.7 and 2.6 in the high-risk and low-risk groups, respectively, which is double-edged. In the literature, emotional distress due to diseases might motivate people to cope positively with the illness and take measures to remove related distress [46]; emotional representation was positively associated with smoking cessation, diet management, and taking medication [47]. However, negative emotional representations were also associated with mental distress [46] and self-stigma [48]. Emotional representations may result in maladaptive coping that could reduce health-promoting behaviors [46]. As interventions promoting OSA detection may attempt to increase Consequences and Timeline chronic, such changes in cognitive illness representations might also create negative emotional representations. To reduce emotional representations, related interventions should explain to high-risk people that OSA treatments are available, safe, and effective.

It was unexpected that Illness coherence was significantly associated with conditional BICDE-OSA in the low-risk, but not high-risk, group. As the high-risk group had a relatively small sample size, it is plausible that the non-significance of illness coherence in this group might be partially attributed to its relatively low statistical power. Future studies should confirm the significance of the association among high-risk individuals and investigate the levels of specific knowledge and attitudes concerning various aspects of OSA that would affect BICDE-OSA.

It is also unexpected that Treatment control was not a significant factor of BICDE-OSA. CPAP is a commonly used, non-invasive, safe, and effective treatment that can be used by OSA patients themselves without external help [7]. The relatively low level of this domain suggests that the public might not be familiar with such effective treatments, and hence, were not motivated by treatment control when deciding to take up OSA diagnostic examination. Health promotion of the effectiveness of OSA treatment is deemed necessary. Personal control was also significantly associated with conditional BICDE-OSA in the low-risk, but not high-risk, group. Statistical power may again be a potential partial reason for the non-significance.

The findings of this study have important practical implications on promoting OSA examination. First, it might be useful to raise the high-risk individuals’ awareness and knowledge of OSA. Several intervention studies have demonstrated that health education programs tailored to an individual’s needs, including inadequate knowledge and mental distress, were effective in increasing OSA detection rates [14, 49–52]. The significant factors found in this study (e.g., Consequence, Timeline chronic/cyclical, and Treatment control) would guide the design of specific contents in related health education programs. Furthermore, effective interventions in the literature include those involving peers and healthcare workers and those conducted in community settings or via telephone [49–53]. Notably, social media could also be an effective platform to scale up these interventions; a qualitative study reported that patients tend to seek information and support from the internet [16]. In addition, free screening may be provided to the older people in community centers and primary care settings, incorporating simple free or low-cost consultations about the importance of performing clinical diagnostic examinations through videos, hotlines, apps, social media, and even artificial intelligence techniques.

Although cognitive changes are important, the socio-ecological model [54] reminds us that structural factors are as important as individual factors. Accessibility to OSA diagnostic services is an important barrier, including long waiting times and high financial costs [14, 23, 55]. For instance, in Hong Kong, a limited number of public hospitals are offering low-cost (about USD25) clinical diagnostic examinations for OSA. Although there has been a slight expansion in the examination services, the average waiting time is still over six months for an OSA examination. Private services, which do not require long waiting times, are available in Hong Kong at the cost of USD 1200–1800; the CPAP costs USD 600–1200; these costs may not be affordable to grassroots people. Thus, given the high prevalence, low detection, and treatability of OSA, the government needs to expand OSA examination services, including subsidies and collaborations with private clinicians and technicians to reduce the out-of-pocket costs. In parallel, people who can afford private services should be recommended to use existing private services to avoid potentially severe consequences of OSA; they should see this as a worthwhile, cost-effective, and necessary health investment.

This study has several limitations. First, it is acknowledged that there is a gap between intention and actual behavior, although the former is a good predictor of the latter [33]. Longitudinal studies investigating whether illness representations of OSA would predict actual OSA examination behavior are needed. Intervention studies testing the effectiveness of changing illness representation of OSA on changes in OSA clinical examination behavior and BICDE-OSA are also warranted. Second, as the prevalence of unconditional BICDE-OSA was low in this study, analysis of its associated factors was not feasible given the study’s sample size. Third, responses to some of the Berlin Questionnaire’s items (e.g., frequency of snoring) may involve recall bias; weight and height were self-reported while obese people tended to under-report body weight in surveys [56]. In addition, since there are no available scales for measuring the BICDE-OSA, such measures were constructed for this study, following the guideline recommended by the original developer of the TPB [57]. Namely [1], defining the behavior of interest clearly in terms of its target, action, context, and time elements, and [2] formulation of items following the examples from the guidelines and previous TPB-based studies [58–60]. Fourth, as people might perceive a social expectation that they should take care of themselves, the prevalence of conditional BICDE-OSA might be inflated because of biased social desirability. Fifth, although the response rate of 55.5% was comparable to those of other local telephone surveys [61], non-respondents might have weaker health awareness and thus lower BICDE-OSA than the participants in this study. As this study only recruited the Chinese adult general population aged ≥ 50 years in Hong Kong, generalization to other populations and regions/countries should be made cautiously. Last, the sample size of the high-risk group was relatively small and may result in relatively low statistical power.

In sum, despite the high prevalence of OSA among people aged ≥ 50 years and the availability of safe/effective OSA treatments, the prevalence of unconditional BICDE-OSA was low. Notably, the prevalence of BICDE-OSA increased by about ten-fold if the high-risk individuals were made aware of their high-risk status of OSA. This study was the first one revealing significant associations between modifiable illness representations of OSA and BICDE-OSA; partial support for the hypotheses was found. Cross-cultural comparisons and validation of the findings in longitudinal and intervention studies would be useful.

## Data Availability

Data is available upon reasonable request from the corresponding author.
